# Assessment of scabies and its associated factors in Hawassa Zuria District, Southern Ethiopia: A cross-sectional study

**DOI:** 10.1371/journal.pone.0314140

**Published:** 2024-11-21

**Authors:** Philmon Dejen, Mekonnen Girma, Adane Chernet, Susana Vaz Nery, Techalew Shimelis

**Affiliations:** 1 School of Medical Laboratory Sciences, College of Medicine and Health Sciences, Hawassa University, Hawassa, Ethiopia; 2 Department of Medical Laboratory Science, Asrat Woldeyes Health Science Campus, Debre Berhan University, Debre Berhan, Ethiopia; 3 School of Medicine, College of Medicine and Health Sciences, Hawassa University, Hawassa, Ethiopia; 4 Kirby Institute, University of New South Wales, Sydney, Australia; MeU: Mattu University, ETHIOPIA

## Abstract

**Background:**

Scabies is a common but neglected skin disease caused by the parasitic mite *Sarcoptes scabiei var*. *hominis*. Globally, the disease affects more than 400 million people. Although Ethiopia is a high-burden country for scabies, its epidemiology has not been well assessed. Therefore, this study aimed to determine the prevalence of scabies, clinical features, and associated risk factors in the communities of the Hawassa Zuria District of the Sidama Region, southern Ethiopia.

**Methods:**

A community-based cross-sectional study was conducted from September through November 2023 in the Hawassa Zuria District. A multistage random sampling technique was applied to enrol 511 participants. The International Alliance for Control of Scabies Diagnostic Criteria was used for examination. The data were collected electronically using the Open Data Kit application through a pretested interviewer-administered questionnaire. Analysis was performed using STATA software. The binary logistic regression analyses model was used to assess the association between each independent variable and scabies prevalence. During the bivariate logistic regression analyses a variable with a p-value of < 0.25 was a candidate for multivariable logistic regression analyses. In multivariable logistic regression analyses the odds ratios with a 95% confidence interval and a p-value less than 0.05 were used to describe the strength of the association and statistical significance.

**Results:**

The median age of the study participants was 19 years (interquartile range: 11–32 years), and 52.6% of the participants were females. The overall prevalence of scabies was 6.3% (95% CI 4.3–8.7%). The majority of individuals with scabies had a moderate degree of severity. The most frequent lesions were intensely itchy papules, vesicles, and pustules that appeared in the interdigital space, flexor wrist surfaces, and elbow. Males were more likely to have scabies [adjusted odds ratio (AOR) = 2.57; 95% CI: 1.10–6.00] than females were and it was not influenced by age. The risk of scabies was higher for households with low (AOR = 3.88; 95% CI: 1.01–14.91) and middle-class wealth index (AOR = 4.43; 95% CI: 1.13–17.33), as well as for individuals residing in households with an overcrowding index >1.5 (AOR = 2.64; 95% CI: 1.13–6.18), in those individuals who washed their hands with water only (AOR = 2.98; 95% CI: 1.23–7.24), in those who used an unimproved water source (AOR = 2.98; 95% CI: 1.25–7.06) and in those who slept on the floor (AOR = 2.70; 95% CI: 1.17–6.18).

**Conclusion:**

The observed moderate presence of scabies in the study area stresses the need to strengthen disease management efforts, improve wealth, reduce overcrowding, ensure clean water access, and promote better hygiene practices to reduce the spread of scabies.

## Introduction

Scabies is a common and highly contagious ectoparasitic infestation of the skin caused by the obligate human itch mite (female), *Sarcoptes scabiei* variety *hominis* [1]. Globally, over 400 million people experience scabies annually [2], accounting for 0.21% of disability-adjusted life years [3]. Scabies mites are spread via direct and prolonged skin-to-skin contact (usually for 15 to 20 minutes) with someone who has scabies [1] or indirectly through contact with infested items like clothing, bedding, and furniture [4]. Asymptomatic individuals can transmit scabies and mites locate hosts using odour and temperature gradients [1]. However, preventive measures, including maintaining personal hygiene, avoiding contact with infected individuals, and treating clothing and bedding, are crucial for controlling the spread of scabies [4, 5].

Scabies significantly impacts quality of life [5, 6], primarily through sleep deprivation, psychological stress, and social isolation, due to intense itching, which worsens at night as the mites burrow into the skin [5–7]. A rash with small red bumps or blisters typically appears in skin folds, such as between finger webs, wrists, axillae, buttocks, abdomen, and genitalia. Burrows may also be visible on the skin’s surface [6]. Scabies also facilitate bacterial infections caused by *Streptococcus pyogenes* and *Staphylococcus aureus*, which can lead to systemic complications including nephritis, rheumatic fever, impetigo, and sepsis [2, 8]. Scabies result in wasted resources on ineffective treatments, lost productivity, and public health costs, making it a significant socioeconomic burden [2, 9]. It has been reported that scabies also impact school performance and lead to increased work absenteeism [10, 11].

In Africa, scabies is a major public health issue, affecting millions due to poverty, overcrowding, war, migration, and limited healthcare access [12, 13]. Prevalence varies significantly, ranging from 71% in Ghana, 17.8% in Cameroon, 2.9% − 39.2% in Malawi, to 4.4% in Egypt [7, 12, 14–16]. The infestation impacts all demographics, with increased risk for children, the elderly, immunocompromised individuals, disabled individuals, and those in tropical areas [3, 8]. Although less common in developed countries [17], scabies still affect economically disadvantaged groups such as street dwellers and migrants [18].

Efforts to mitigate scabies in Africa require a comprehensive approach that addresses early diagnosis, treatment, and prevention [7–9]. Diagnosing scabies is challenging, as it relays the clinical history and physical examination, which lack specificity [5, 19, 20]. The gold standard method to confirm scabies involves microscopic examination of skin scrapings, which is less sensitive and often inaccessible [20]. This can lead to misdiagnosis and treatment delays, allowing scabies to spread rapidly in communities with poor hygiene and sanitation [5, 19]. Mass drug administration (MDA) programs with permethrin or oral ivermectin have shown promise in high-burden areas but limited healthcare resources and infrastructure often hinder effective implementation often hinder effective implementation [7]. In Zanzibar, ivermectin administration for lymphatic filariasis control led to a 68–98% reduction in scabies cases [9].

In Ethiopia, scabies pose a significant public health challenge [21, 22], with outbreaks influenced by the country’s vulnerability to climate variability and change [23]. Some studies have shown that the prevalence of scabies in Ethiopia ranges from 11–33.5% [22–25], with a higher burden observed in areas with limited access to clean water, high population mobility, internal displacement, and insufficient healthcare infrastructure [26], Conditions such as drought, malnutrition, and limited interventions exacerbate the spread and severity of scabies [22]. It is also common during natural or man-made disasters like flooding, civil war, and violence [24].

Despite its significance, epidemiological studies on scabies in Ethiopia are limited. Existing data are often restricted to specific institutions or outbreaks and specific age groups with limited generalizability, and provide minimal insight into risk factors [27]. Additionally, These studies rely mainly on clinical examinations of limited body parts [10, 24, 26, 28], which may underestimate prevalence by up to 10%, while reliance on clinical signs may have led to misclassification and overestimation [19]. Since 2015, the Ethiopian Federal Ministry of Health has partnered with various organizations to combat scabies transmission, particularly in high-risk communities [4]. Reliable epidemiological data are essential for designing effective intervention strategies [16], and community-based data are considered good proxies for understanding prevalence [16, 22]. This study, therefore, aims to assess the prevalence, clinical features, and associated factors of scabies in the communities of Hawassa Zuria District, southern Ethiopia.

## Materials and methods

### Study design and setting

**Fig 1:**
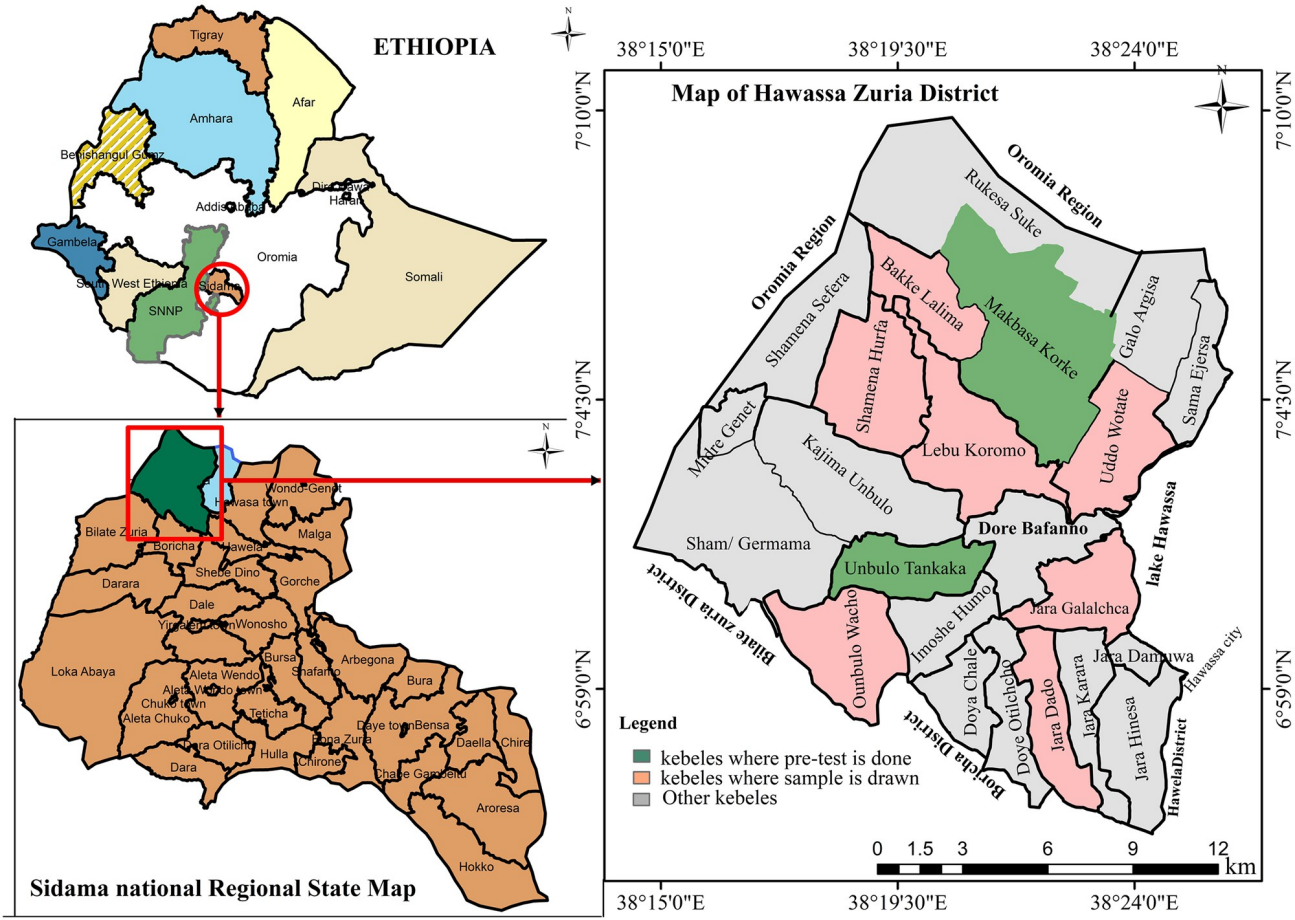
Map of the study area (Hawassa Zuria District), Sidama Region, southern Ethiopia, 2023 [prepared using ArcGIS 10.8 (ESRI, Redlands, USA) after obtaining country shape files from the (humdata.org) website].

A community-based cross-sectional study was conducted from September 23 to November 10, 2023, in Hawassa Zuria District, Sidama Region, southern Ethiopia. The district is located 21 km west of Hawassa city, the regional capital, and approximately 290 km south of Addis Ababa, the capital city of Ethiopia (Fig 1). In 2022, the estimated district’s total population was 178,905, with 90,347 (50.5%) females and 88,558 (49.5%) males, with 36,511 households. The district has a total land area of 305.24 km^2^ and is divided into 23 administrative *kebeles* (the smallest administrative unit in Ethiopia, with an average population of 4,000 living in each *kebele*). It has four public health centres, twenty-three health posts, one primary hospital, and eight private clinics that serve the entire population [29]. The district was selected purposively considering that the frequency of scabies reported to the Sidama Region Public Health Institute in 2022 was greater in the district than in other districts. In addition, malnutrition, infectious diseases, and limited access to clean water, which worsen the burden of scabies, are further issues in this area.

### Population

The source population consisted of all residents of the Hawassa Zuria District, and the study populations were residents of each chosen *kebele* within the district. All individuals who were permanent residents within the last six months in each selected *kebeles* of the Hawassa Zuria District during the study period were included in this study. However, household members who were not present at home during the data collection time and individuals who were taking scabicide medication within the last two weeks were excluded. However, those selected household members who either declined to participate withdrew from the study, or failed to provide a sample were categorized as nonrespondents.

### Sample size determination

The sample size required for the study was estimated using the Epi Info^™^ version 7.2.5.0 (Centers for Disease Control, Atlanta, USA) statistical program. The sample size for the assessment of scabies prevalence was calculated to be 526 using a single population proportion formula with the assumptions of a 19.3% prevalence of scabies [25], a 95% confidence level, 5% precision, a design effect of 2, and a 10% nonresponse rate.

### Sampling technique

As shown in S1 Fig, a multistage sampling technique was employed. Initially, the district, which is divided into five catchment areas, each containing 4–5 *kebeles*, was purposefully selected. Next, three catchment areas were randomly selected. Seven *kebeles* were then nominated by the lottery method, in proportion to the number of *kebeles* in each catchment representing 30% of the district’s total 23 *kebeles*. This method aimed to ensure the sample’s representativeness. The selected *kebeles* were Unbulo Wacho, Shame Hurfa, Bekke, Jara Galacha, Ouddo Wotate, Jara Dado, and Labu Kormo. Then the sample size was proportionally allocated to each selected *kebele* according to the number of households. Households in each *kebele* were selected using a systematic sampling technique, with the household list serving as the sampling frame. The sampling interval (K) was 22, calculated by dividing the total households (n = 11,523) in the seven selected *kebeles* by the sample size (n = 526). A random selection between 1 and 22 determined the first household, with every 22^nd^ household selected thereafter. One person from each household was chosen using a simple random sampling technique. If a house was closed during the first visit, data collectors returned. If it remained closed on the second visit, the nearest house was used as a substitute.

### Data collection

#### Interview

The data were collected by two experienced and trained health officers (a cadre with 4 years of training in clinical and public health services). Data collectors conducted a face-to-face interview at the selected house and collected data using structured questionnaires (S1 Appendix). If individuals aged eighteen and above were selected, they were interviewed directly. For those under eighteen years old, the head of the household or caregiver was interviewed on their behalf.

Data about sociodemographic (e.g., age, sex), socioeconomic (e.g., wealth index), and environment-related (e.g., climatic condition, overcrowding index, source of water) factors were gathered through interviews. Furthermore, participants were interviewed about behaviours such as sharing clothes and a history of contact with a scabies-infested person, as well as the practice of maintaining personal hygiene, like washing hands with soap or detergent. Each criterion was assigned a value of “1” or “0” to indicate the presence or lack of hygienic practice, respectively. The calculated sum and the mean score of all observations were utilized as cut-off points to classify personal hygiene practices as good or poor. A score higher or lower than the mean value was considered a good or poor personal hygiene practice, respectively.

Comprehensive knowledge of the head of the household regarding scabies was assessed by asking general questions that focused on the aetiology of scabies, identification of the signs and symptoms of scabies, its characteristics, modes of transmission, prevention, and control measures. The correct or incorrect response for each item received a score of "1" or "0", respectively. Total scores were calculated and interpreted based on a previous study [11] by summing the participants’ scores across all knowledge questions, and the score was categorized into two levels. Respondents were considered to have "good” or “poor” knowledge if the mean value of the sum of the knowledge assessment questions was higher or lower, respectively.

The attitude of the head of the household towards scabies infection was assessed by a five-point Likert scale [30]. The questions on the Likert scale had positive and negative responses that scored five to one. The attitude score was estimated by summing the scores across the items used to measure attitude and was categorized based on the median of the total score. Participants who scored greater than or equal to the median score were considered to have a positive attitude, while those with a score below the median score were considered to have a negative attitude towards scabies [31]. All the data were recorded on an Android smartphone using a customized electronic data capture tool called the Open Data Kit (ODK)platform.

#### Clinical examination

Participants were examined by two health officers, and the diagnosis was ascertained based on the classification and case definition criteria set by the IACS (Table 1). In the current study, scabies was diagnosed if the investigator identified one of the following subcategories: A1, B1, B2, B3, C1, or C2. Subcategories A2 and A3 were not considered in this study due to constraints in accessing examination instruments [5].

**Table 1:** The 2020 international alliance for the control of scabies consensus criteria for the diagnosis of scabies [5].

Category	Sub-category	Description	Used in this Study
Confirmed scabies	A1	Mites, eggs, or faeces on light microscopy of skin samples	Yes
	A2	Mites, eggs, or faeces visualized on an individual using a high-powered imaging device	No
	A3	Mite visualized on an individual using dermoscopy	No
Clinical scabies	B1	Scabies burrows	Yes
	B2	Typical lesions affecting male genitalia	Yes
	B3	Typical lesions in a typical distribution and two history features ^a^	Yes
Suspected scabies	C1	Typical lesions in a typical distribution and one history feature ^a^	Yes
	C2	Atypical lesions or atypical distribution and two history features ^a^	Yes

^a^ history features are itch and positive contact history.

The severity of the infestation was estimated by counting the number of lesions, with classification criteria adapted from a previous study [19], as scabies severity was defined and categorized as mild (1–10 lesions), moderate (11–49 lesions), or severe (≥50 lesions).

#### Microscopic examination

This was performed for those who fulfilled clinical and suspected case definitions. Two experienced and trained medical laboratory technologists with a 4-year training degree collected samples from the lesion using adhesive tape and transferred them onto microscopic slides. The slides were transported to the nearest health facility by motorbike in a sample transporter cold box and examined microscopically within five minutes of collection [20, 32]. The presence of eggs, immature and adult mites, or scybala (faeces) is reported as a confirmed diagnosis of scabies [5, 20, 32].

### Study definitions

**Household** can be defined as a person or group of related or unrelated persons who live together in the same dwelling unit(s), who acknowledge one adult male or female as the head of the household, who share the same housekeeping arrangements, and who are considered a single unit [33].

**Contact** is defined as a person who has prolonged, direct skin-to-skin contact, individuals who share a bed, including sexual partners, children in the same classroom, or who play closely together with a suspected, clinical, or confirmed case in the last two months before the survey. It is expected that a single infected person may contact around five individuals [4, 5].

**Improved water sources** are defined as those that are likely to be protected from outside contamination and faecal matter in particular. It includes whether the community obtained water from protected springs, protected wells, public taps, or rainwater. An "unimproved" water source includes if the community obtained water from unprotected springs or unprotected wells, tanker truck-provided water and surface water (river/dam/lake/pond/stream/canal/irrigation channel), unprotected springs, or unprotected wells [33–35].

**Hand washing practice**: If an individual consistently washes their hands with soap and water before a meal, after meals, before food preparation, after visiting the toilet, and after handling rubbish or animals, it is considered to be in adherence to “good hand hygiene practices”. On the other hand, if an individual washes without soap (water only), it is considered poor adherence [11].

**Improved sanitation facilities:** are defined as those that hygienically separate human waste from human contact. It includes a flush toilet, a ventilated improved pit latrine, and a pit latrine with a slab. Also, Pit latrines without a slab or open pit, bucket latrines, hanging latrines, and no facility/bush /field are not considered to be improved sanitation [33, 34].

**Bathing** is defined as individuals who wash their body less than once per week in the past month are categorized as having an "infrequent" bathing practice. Conversely, those who engage in washing their body more than once per week are identified as having a "frequent" bathing practice [23].

**Clothes washing/ changing practice:** Participants who wash their clothes less than once per week in the past month are identified as having an "infrequent" washing of clothes. Conversely, individuals who engage in washing their clothes more than once per week are classified as having a "frequent" washing of clothes. Likewise, the cloth-changing practice is considered "infrequent" if they change their clothes less than once per week in the past month. On the contrary, individuals who change their clothes more than once per week are considered to have a "frequent" changing of clothes clothes [23].

**Access to water** is categorized based on the daily per capita water collection. If households collect below 30 liters per capita per day (l/c/d), it is considered "no access." Conversely, if households collect more than 30 l/c/d of water, it is categorized as "basic access." Similarly, the distance of the water source is defined as a "limited water service" if the round trip to collect water, including queuing, exceeds 30 minutes. On the other hand, if the entire process takes no more than 30 minutes it is considered as a “basic service” [35].

### Data analysis

An Arc map was used for mapping the distribution of cases and the administrative area of the study. The ODK-based collected data were extracted from were extracted from the Kobo toolbox server and checked for completeness and consistency. The cleaned data were exported into STATA software for Windows version 16.1 (Stata Corp, College Station, Texas 77845 USA, 2019) for analysis. Descriptive results are presented as frequencies, percentages, medians, and interquartile ranges. Potentially significant independent predictors of scabies at a p-value of less than 0.25 in the bivariate analysis were included in a multivariable logistic regression analysis to compute the adjusted odds ratio (AOR). A p-value less than 0.05 was considered to indicate statistical significance. Multicollinearity was checked using the variance inflation factor (VIF ≤ 10), and the Hosmer and Lemeshow goodness-of-fit test (p > 0.05) was applied to check the adequacy of the final model [36].

We employ the wealth index, which is a composite measure of a household’s cumulative living standard, determined through principal component analysis (PCA). Household ownership of selected assets (items, livestock, vehicles, materials used for dwelling construction, access to specific services, and agricultural land ownership) was assessed. To conduct PCA, essential variables were explored using the rule of thumb, which suggests excluding variables owned by more than 95% or less than 5% of the sample. Moreover, a median was computed for all non-dummy variables for input in PCA, with those scoring equal to or above the median coded as “1” and those scoring below the median coded as “0”. PCA assumptions such as the Kaiser–Meyer–Olkin test (KMO) for sampling adequacy (yielding a value of 0.67) and Bartlett’s test of sphericity (statistically significant at a p-value < 0.001) were checked. A correlation matrix was also checked, and there were more than two variables with a correlation value ≥ 0.3. Variables with a commonality value less than 0.5 were excluded before generating the final factor scores, leaving 19 variables eligible for factor scoring. The score for each household on the first principal component was retained to create the wealth score. These scores were then divided into three equal population quintiles: lowest, middle, and highest [33, 37]. The overcrowding index was determined based on previous literature [16, 31] by dividing the number of regular residents by the number of bedrooms in the house. A value > 1.5 indicates that the house is overcrowded; a value ≤ 1.5 indicates that the house is not overcrowded.

### Ethics approval and consent to participate

This research was carried out after obtaining ethical approval from the Institutional Review Board (IRB) of the College of Medicine and Health Sciences of Hawassa University (Reference number IRB/203/15). The respondents’ participation was fully voluntary. Written informed consent was obtained from participants aged 18 and above, as well as from parents/guardians of children under 12 years old. For children aged 12 to less than 18 years, both assent from the child and written informed consent from their parents/ legal guardians were obtained. Each participant was assigned a unique identifier to maintain the confidentiality of the information. Individuals diagnosed with scabies were treated following standard local protocols. All methods were carried out following relevant guidelines and regulations.

## Results

### Sociodemographic and socioeconomic characteristics of participants

This study enrolled a total of 511 households, with one participant from each household, achieving a response rate of 97.2%. There were 15 individuals (2.8%) who refused to participate. The median age of the study participants was 19 years with an interquartile range (IQR) of 11–32 years. Approximately half of the participants (49.1%) were under the age of 18 years. All participants resided in rural areas, and 52.6% of the participants were female. A significant proportion of the study participants (56.4%) had attained a primary education, while the predominant occupational group was students (41.5%), followed by farmers (25.2%). One-third (33.4%) of the participants were from low-income households (Table 2).

**Table 2:** Sociodemographic and socioeconomic characteristics of the participants in Hawassa Zuria District, Sidama Region, southern Ethiopia, 2023.

Variables	Category	Frequency (%) (N = 511)
Gender	Male	242 (47.4)
	Female	269 (52.6)
Age (years)	<5	46 (9.0)
	5–9	58 (11.4)
	10–14	96 (18.8)
	15–24	116 (22.6)
	25–34	76 (14.9)
	35–44	57 (11.2)
	≥45	62 (12.1)
Educational status	No formal education	148 (29.0)
	Primary school	288 (56.4)
	Secondary & above	75 (14.6)
Occupation	Student	212 (41.5)
	Farmer	129 (25.2)
	Housewife	69 (13.5)
	Unemployed	81 (15.9)
	employed	20 (3.9)
Wealth index	Poor	171 (33.4)
	Middle	170 (33.3)
	Rich	170 (33.3)

### Scabies prevalence and classification

Among the 511 participants, 32 were diagnosed with scabies, with an overall prevalence of 6.3% (95% CI 4.3–8.7%). According to IACS classification criteria, 21.9% of the 32 scabies cases were confirmed scabies (subcategory A1), 34.3% of cases were clinical scabies subcategory B3 (typical lesions in a typical distribution and two history features), 9.4% of clinical scabies subcategory B2 (typical lesions affecting male genitalia); 3.1% were clinical scabies subcategory B1 (scabies burrows); 25% of cases were suspected scabies subcategory C1 (typical lesions in a typical distribution and one history feature), and 6.3% were suspected scabies subcategory C2 (atypical lesions or atypical distribution and two history features).

**Fig 2:**
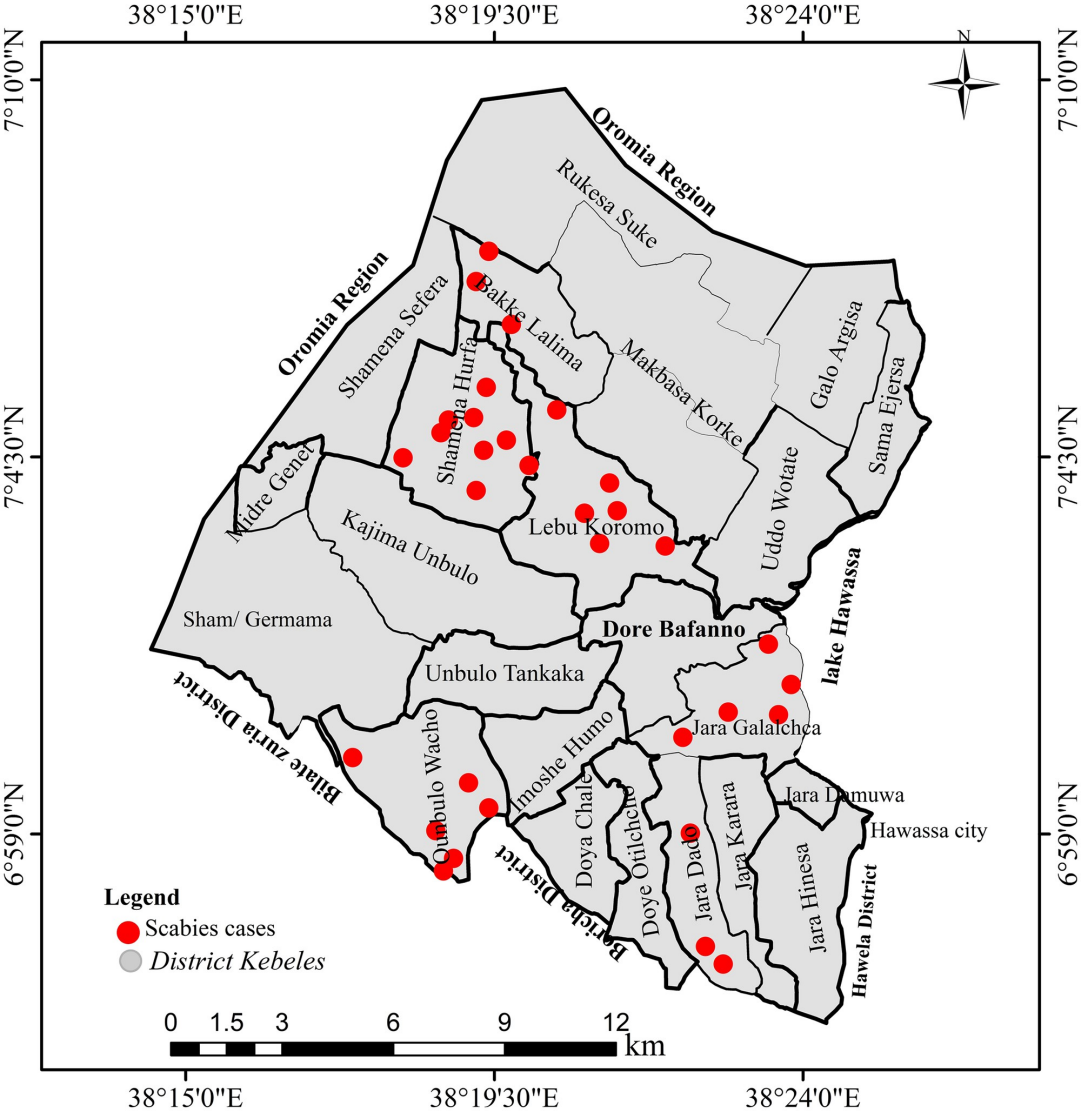
Spatial patterns of kebele-level distributions of scabies in Hawassa Zuria District, Sidama Region, southern Ethiopia, 2023.

Among the 32 individuals with scabies, 9 (28.1%) were from Shamena Hurfa *Kebele*, 6 (18.8%) were from Labu Kormo, 6 (18.8%) were from Unbulo Wacho *kebeles*, and no scabies was found in the Uddo Wotate *kebele*. The *kebele*-specific prevalence of scabies was 10.5%, 7.3%, 6.7%, 6.3%, 5.9%, and 5.2% in Shamena Hurfa, Unbulo Wacho, Bekke Lalima, Jara Galacha, Labu Kormo, and Jara dado, respectively. Fig 2 shows the spatial distribution map created based on the geographic coordinates of the scabies that were gathered using the Global Positioning System.

### Scabies severity and clinical features

Of 32 scabies cases, the majority (71.9%) exhibited moderate severity, while 15.6% and 12.5% had mild and severe scabies, respectively. Itching was the most common clinical symptom, identified in 96.9% of cases, with 90.6% experiencing severe itching during the night. Additionally, scabies-associated signs such as skin rash were observed in 40.6% of cases, and 53.1% of cases presented with a secondary bacterial infection (Table 3).

**Table 3:** Scabies severity and clinical features among cases in Hawassa Zuria District, Sidama region, southern Ethiopia, 2023.

Variable	Category	Frequency (%) (N = 32)
Scabies severity	Mild (<10 lesions)	5 (15.6)
	Moderate (11–49 lesions)	23 (71.9)
	Severe (≥50 lesions)	4 (12.5)
Scabies associated symptoms	Skin itch (pruritus)	31 (96.9)
Observed scabies-associated signs	Skin rash	13 (40.6)
	Tiny red burrow	2 (6.3)
	Secondary bacterial infection	17 (53.1)
Time of itching	Severe during night	29 (90.6)
	Severe during day	2 (6.3)
	Similar in both day and night	1 (3.1)

**Fig 3:**
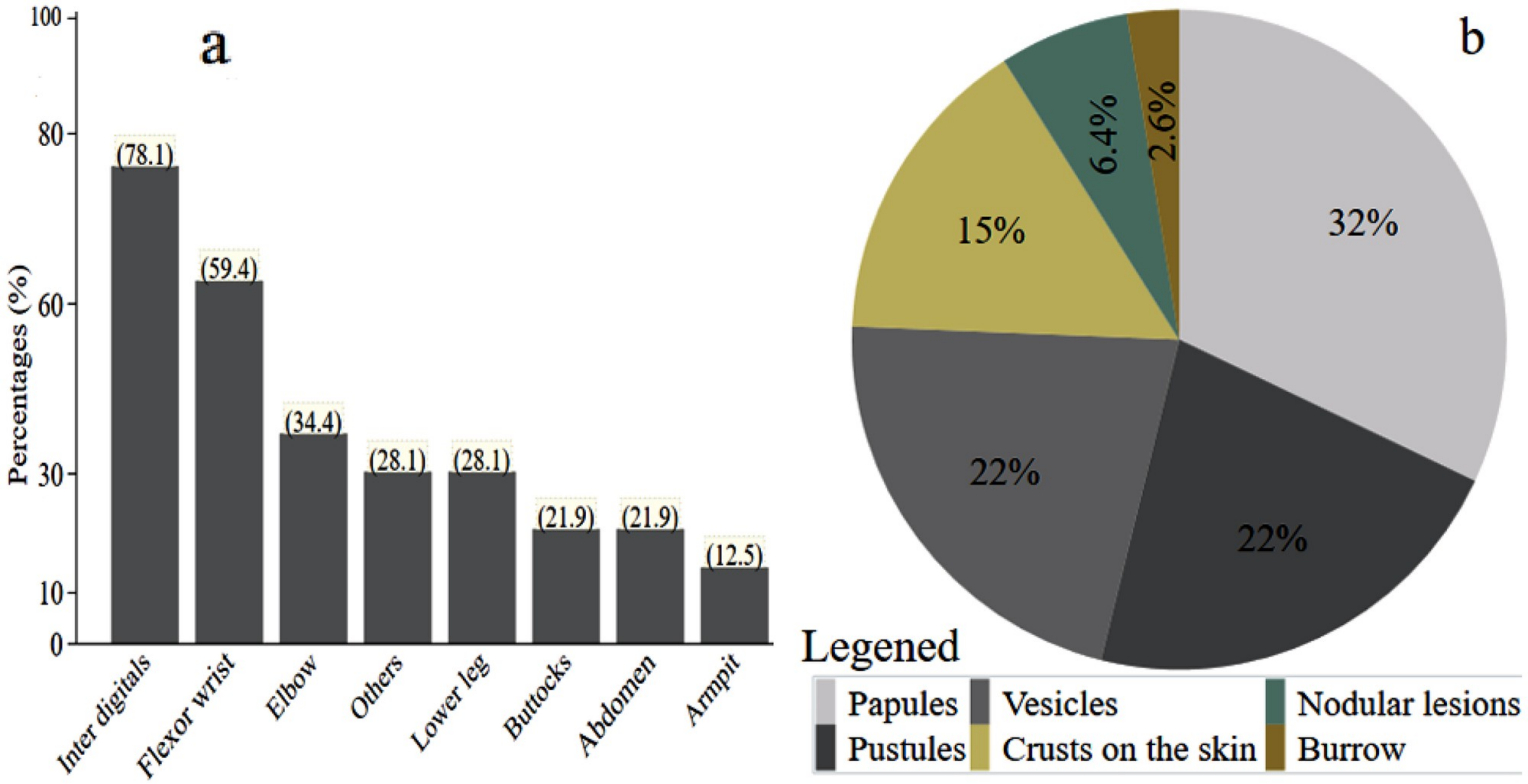
Anatomical location of lesions in individuals infected with scabies (a) and types of scabies-related skin lesions (b) in Hawassa Zuria District, Sidama Region, southern Ethiopia, 2023. Others: Thigh (2), breast (1) genital (3) shoulder (2), and head (1).

Among individuals who presented scabies lesions, the most frequently observed anatomical sites of infestation were the interdigital space (78.1%), flexor wrist surface (59.4%), and elbow (34.4%) (Fig 3a). The predominant types of lesions identified were papules (32%), followed by vesicular and pustular skin lesions, each observed in 22% of cases (Fig 3b).

### Home environment-related characteristics

In this study, it was found that 40% of 511 participants resided in housing conditions characterized by overcrowding. Participants also reported environmental challenges, with 32.5% mentioning that there was flooding in the vicinity of their residence, and 16.3% of them reported that their homes had been affected by flooding. A total of 69.3% of participants had health facilities built near their homes and 48% of participants obtained water by traveling for more than 30 minutes. Geographically, the majority (67.7%) of participants resided in the lowland part of the district. Furthermore, 39.1% of participants reported having pet animals inside their homes, with cats (49.5%) being the most common animal (Table 4).

**Table 4:** Home environment-related characteristics of the participants in Hawassa Zuria District, Sidama Region, southern Ethiopia, 2023.

Variables	Category	Frequency (%) (N = 511)
Overcrowding index	≤1.5	307 (60.0)
	>1.5	204 (40.0)
The presence of flooding in your area	No	345 (67.5)
	Yes	166 (32.5)
Homes affected by Flooding (N = 166)	No	139 (83.7)
	Yes	27 (16.3)
Distance of health facility from the household	Near (≤1 hour)	354 (69.3)
	Far (>1 hours)	157 (30.7)
The climatic condition of the participant’s residence site	Lowland	346 (67.7)
	Midland	165 (32.3)
Distance of water source from household	Basic service (≤30’)	266 (52.0)
	Limited service (>30’)	245 (48.0)
The presence of pet animals in the house	No	311 (60.9)
	Yes	200 (39.1)
Type of animal (N = 200)	Cat	99 (49.5)
	Dog	76 (38.0)
	Other	25 (12.5)

### Personal hygiene and behavioural-related characteristics

Of the study participants, 55.4% did not have access to health information on personal hygiene. However, 58.3% of the participants practiced washing their hands with soap and water. Furthermore, 59.5% of participants obtained a sufficient amount of water for their households, and 65.6% of participants had access to water from improved sources. In addition, 61.5% reported taking frequent baths, 49.1% reported washing their clothes frequently, 53% reported changing clothes frequently, 57.1% reported washing their hair frequently, and 63.3% reported trimming their fingernails during the time of data collection. Regarding behavioural-related characteristics, 62.4% of participants slept on a bed. Three-fourths (75.9%) of the participants shared a bed with other family members. The practice of sharing clothes with individuals infected with scabies was reported by 2.9% of participants. Similarly, skin contact with scabies cases was reported in 6.5% of participants, with 39.4% having contact with their family members. In addition, 66.7% of participants practice drying clothes and bedding. Overall, 66.8% of the participants had good knowledge about scabies. According to the overall attitudes score, more than two-thirds of the participants (70.1%) had a positive attitude towards scabies (Table 5).

**Table 5:** Personal hygiene and behavioural-related characteristics of the participants in Hawassa Zuria District, Sidama Region, southern Ethiopia, 2023.

Variables	Category	Frequency (%) (N = 511)
Access to health information on personal hygiene	Yes	228 (44.6)
	No	283 (55.4)
Participant’s hand-washing practice with	Soap with water	298 (58.3)
	Water only	213 (41.7)
Approximate amount of water accessed by households	Insufficient (≤30 litre)	207 (40.5)
	Sufficient (>30 litre)	304 (59.5)
Source of water for personal hygiene	Improved source	335 (65.6)
	Unimproved source	176 (34.4)
Bathing frequency	Frequent	314 (61.5)
	Infrequent	197 (38.5)
Clothes washing frequency	Frequent	251 (49.1)
	Infrequent	260 (50.9)
Frequent changing of clothes	Yes	271 (53.0)
	No	240 (47.0)
Hair washing frequency	Frequent	292 (57.1)
	Infrequent	219 (42.9)
Fingernail trimming	Yes	323 (63.3)
	No	188 (36.7)
Personal hygiene practice	Good	239 (46.8)
	Poor	272 (53.2)
Sleeping place	On the bed	319 (62.4)
	On the floor	192 (37.6)
Bed sharing	No	123 (24.1)
	Yes	388 (75.9)
Sharing of clothes with scabies patient	No	496 (97.1)
	Yes	15 (2.9)
Contact with a scabies-infested person	No	478 (93.5)
	Yes	33 (6.5)
Relationship with the contact person	Family member	13 (39.4)
	Neighbours	11 (33.3)
	Others	9 (27.3)
A habit of drying clothing/bedding in the sun	Yes	341 (66.7)
	No	170 (33.3)
Mean knowledge score	Good knowledge	326 (66.8)
	Poor knowledge	162 (33.2)
Overall attitude	Negative attitude	146 (29.9)
	Positive attitude	342 (70.1)

### Scabies risk factors

Among the variables that were assessed for their association with scabies in the bivariate logistic regression analysis, the candidates for multivariable logistic regression analyses were gender (p = 0.015), age (p = 0.245), wealth index (p = 0.006), overcrowding index (p = 0.001), climatic condition of the participant’s residence site (p = 0.030), access to health information on personal hygiene (p = 0.121), hand washing practice (p = 0.016), source of water for personal hygiene (p = 0.130), bathing frequency (p = 0.173), sleeping place (p = 0.004), cloth sharing with scabies cases (p = 0.039), having contact with scabies case (p = 0.037), and having a family member with scabies (p = 0.050) (S1 Table).

In multivariable logistic regression analysis, the odds of having scabies were significantly higher among males (AOR = 2.57; 95% CI: 1.10–6.00, p = 0.029) than females, and the distribution of scabies was not influenced by age. Moreover, the odds of having scabies were higher among individuals belonging to households with low (AOR = 3.88; 95% CI: 1.01–14.91, p = 0.048) or middle-class wealth index households (AOR = 4.43; 95% CI: 1.13–17.33, p = 0.032) than among those belonging to households with high wealth index households. The likelihood of having scabies was higher among households with an overcrowding index greater than 1.5 (AOR = 2.64; 95% CI: 1.13–6.18, p = 0.024) compared to those in households with an overcrowding index less than or equal to 1.5. Individuals who used only water for handwashing (AOR = 2.98; 95% CI: 1.23–7.24, p = 0.016) were more likely to be infested by scabies than individuals who washed their hands with water and soap. Individuals who obtained water from unimproved sources had almost three times higher odds of having scabies (AOR = 2.98; 95% CI: 1.25–7.06, p = 0.013) than those who obtained water from improved sources. Sleeping on the floor increased the odds of scabies infestation by 2.7 times (AOR = 2.70; 95% CI: 1.17–6.18, p = 0.019) compared to sleeping on a bed (Table 6).

**Table 6:** Multivariable logistic regression analysis of selected factors associated with scabies in the Hawassa Zuria District, southern Ethiopia, 2023.

Variables	Category	Scabies diagnosis		AOR (95% CI)	P value
		Yes (%)	No (%)		
Gender	Female	10 (4.0)	259 (96.0)	1	
	Male	22 (8.7)	220 (91.3)	2.57 (1.10–6.00)	0.029*
Age	<5	3 (6.5)	43 (93.5)	1	
	5–9	5 (8.6)	53 (91.4)	0.84 (0.16–4.42)	0.842
	10–14	8 (8.3)	88 (91.7)	1.32 (0.27–6.41)	0.725
	15–24	9 (7.8)	107 (92.2)	1.12 (0.24–5.16)	0.880
	25–34	3 (4.0)	73 (96.0)	0.43 (0.07–2.70)	0.373
	35–44	1 (1.8)	56 (98.2)	0.32 (0.02–3.82)	0.216
	≥45	3 (4.8)	59 (95.2)	0.69 (0.11–4.30)	0.492
Wealth index	High	3 (1.8)	167 (98.2)	1	
	Middle	17 (10.0)	153 (90.0)	3.88 (1.01–14.91)	0.048*
	Low	12 (7.0)	159 (93.0)	4.43 (1.13–17.33)	0.032*
Overcrowding index	≤1.5	10 (3.3)	297 (96.7)	1	
	>1.5	22 (10.8)	188 (89.2)	2.64 (1.13–6.18)	0.024*
The climatic condition of the participant’s residence site	Low land	16 (4.6)	330 (95.4)	1	
	Midland	16 (9.7)	149 (90.3)	1.97(0.83–4.65)	0.122
Access to health education	Yes	10 (4.8)	218 (95.2)	1	
	No	22 (7.8)	261 (92.2)	1.37 (0.57–3.30)	0.476
Hand washing practice	Soap and water	12 (4.4)	286 (95.6)	1	
	With water only	20 (9.4)	193 (90.6)	2.98 (1.23–7.24)	0.016*
Source of water for personal hygiene	Improved	17 (5.1)	318 (94.9)	1	
	Unimproved	15 (10.1)	161 (89.9)	2.98 (1.25–7.06)	0.013*
Sleeping place	On the bed	12 (3.8)	307 (96.2)	1	
	On the floor	20 (10.4)	172 (89.6)	2.70 (1.17–6.18)	0.019*
Bathing frequency	Frequent	16 (5.1)	298 (94.9)	1	
	Infrequent	16 (8.5)	181 (91.5)	1.50 (0.65–3.43)	0.332
Cloth sharing with scabies cases	No	29 (5.9)	467 (94.1)	1	
	Yes	3 (20.0)	12 (80.0)	1.43 (0.20–10.25)	0.718
Contact with scabies case	No	27 (5.7)	451 (94.3)	1	
	Yes	5 (15.2)	28 (84.8)	1.64 (0.37–7.30)	0.513
Family member with scabies	No	28 (5.8)	458 (94.2)	1	
	Yes	4 (16.0)	21 (84.0)	1.76 (0.39–7.79)	0.455

* Statistically significant at p-value < 0.05, AOR: adjusted odds ratio, CI: confidence interval

## Discussion

This study found a 6.3% (95% CI 4.3–8.7%) prevalence of scabies in the Hawassa Zuria District. Scabies prevalence was higher among males, households with a low or middle wealth index, households with an overcrowding index greater than 1.5, individuals who practised hand washing with water only, individuals who used an unimproved water source, and individuals who slept on the floor. The prevalence of scabies in this study contrasts with other community-based studies in Ethiopia, which show higher (11–33.5%) [22–25] and lower (2.5%) [28]. These differences may be due to varying climatic conditions, socioeconomic factors, and prevention efforts. Study findings during droughts [22, 24] or dry seasons [23, 25] suggest a possible influence of climate on the prevalence of scabies, with mite survival and replication being impacted [38, 39].

The majority of the cases were clinical scabies, subcategory B3, followed by suspected scabies, subcategory C1, as also reported in studies in Ethiopia [25] and elsewhere [19, 40]. The severity of scabies is related to the number of mites on the skin and the duration between infestation and diagnosis [5]. Our findings were in agreement with those from Ghana [14] and Ethiopia [25], in which moderate severity was common. However, research in Liberia [40] found a high proportion of severe cases, while school surveys in Solomon Island [19] and Ethiopia [11] reported a high proportion of mild cases. The main observed lesions were papules, vesicular, and pustular lesions, which is consistent with the findings in Cameroon [15]. However, a study in Nigeria [12] reported a higher proportion of excoriations. These differences may stem from the varying immune status of individuals and the presence of other comorbidities [41], as vesicular and pustular lesions can result from bacterial infections, while nodular lesions may arise from immune responses to mite components [6].

Scabies lesions were often found in the interdigital space, flexor wrist surfaces, and elbow, as reported in studies from Ethiopia [26, 28] and other countries [15, 42]. These may be related to handling mite-contaminated materials or touching scabies-infested individuals. Furthermore, mites may prefer delicate and folded areas, potentially attracted by lipid composition [43]. Scabies often lead to secondary skin infections [5, 44], as clinically diagnosed in half of the cases in our study. As others have noted [19, 24], our observation of nocturnal skin itch as the most common symptom is very suggestive of the presence of scabies.

The preponderance of scabies among males compared to females in the current study was compatible with findings from Ethiopia [10, 25] and other countries [15, 19, 40]. However, it was also reported that scabies was more common among females [31, 42], or that there was no association with gender [11, 16, 28]. These findings may be related to gender-specific variations in the distribution of risk factors for scabies. As shown by our study, males were found to have higher exposure to certain risk factors, like hand washing with water alone, more physical contact, and sleeping on the floor.

Participants in low- or middle-wealth households were about fourfold more likely to have scabies compared to those in higher-wealth households, as reported in other studies in Ethiopia [23, 37]. This may be due to individuals in economically disadvantaged areas struggling to afford medical care and treatment, leading to the increased spread of scabies. Additionally, limited access to proper nutrition compromises immunity, increasing susceptibility to infestations [19, 41].

In this study, living in overcrowded homes increased the odds of having scabies, as reported in other studies [12, 23, 45]. This highlights the importance of overcrowding in the transmission of scabies by promoting contact between household members [41]. However, some other studies have shown no significant influence of overcrowding [16, 46] or even its protective role [31]. This might be due to cultural differences in living conditions and variations in measures for reducing overcrowding.

Participants using unimproved water sources had greater odds of having scabies than those utilizing improved water sources, as also reported in another study [27]. The reason is that limited access to clean water makes it difficult to practice appropriate personal hygiene and sanitation. Furthermore, the inability to use soap or other detergents during hand washing increased the odds of scabies, which is consistent with studies from Ethiopia [11, 25–27, 47] and other country [12]. However, contrasting findings in other reports of no impact of personal hygiene or handwashing with detergents [41] or rubbing with sanitizers on the viability and number of scabies mites [48] require further research to explore the susceptibility of scabies mites to various hand-washing materials.

The greater risk of scabies among individuals who sleep on the floor compared to those who sleep on a bed aligns with other studies [12, 47], although another study [11] found no significant association between scabies and sleeping places. The observed disparity may be due to the different types of flooring used in various communities, some of which provide a conducive environment for mite survival.

Age has been identified as a significant risk factor for scabies infestation. Previous studies show children under 15 years are commonly affected [24, 28]. However, our findings showed that scabies was not influenced by age, as shown in other studies in Ethiopia [25, 47] and Cameroon [15]. Behavioral factors significantly influence scabies infestation. While some studies [12, 23, 25, 46] link sharing clothes with scabies patients to increased risk, others [8, 11, 24] find no such association, aligning with our study. This may be due to *Sarcoptes scabiei* mite being highly susceptible to dehydration when outside the host and it is almost immobile at lower temperatures [1, 8, 38]. Additionally, contact with scabies cases [10, 16, 23, 26] and individuals with itchy skin [11, 26] is often associated with scabies, though our study found no significant association. This discrepancy might be due to differences in study populations and scabies severity, with moderate cases being less contagious than severe ones [49].

This study has some limitations. First, there may be recall and information bias as participants were interviewed about past experiences. Second, cases might have been either over- or under-detected due to the inability to use more sensitive methods, such as dermoscopy, and lack of specificity in clinical diagnosis. Third, cultural and socioeconomic differences may exist, and the 7 selected *kebeles* may not fully represent all 23 *kebeles*. Last, the study looked at one season (spring), so it could not demonstrate how scabies patterns can vary among seasons. Despite these limitations, our findings might be more widely applicable because the study involved individuals of all ages and was conducted in a community. To reduce recall and information bias, we used shorter recall periods, memory aids, standardized questionnaires, and well-trained data collectors. To improve scabies diagnosis accuracy, we employed two health officers who followed standard case definitions, with additional support from the laboratory. To minimize the impact of non-homogeneity among *kebeles*, we used a lottery method to select catchment areas and to choose *kebeles* within each catchment. Future research should include improved diagnostic tools, account for seasonal variations, and cover large areas to enhance the understanding of scabies epidemiology.

## Conclusion

This study revealed a moderate prevalence of scabies in the Hawassa Zuria District and highlights the need for strengthening scabies control strategies. Specifically, it is important to improve wealth, reduce overcrowding, improve access to clean water, and promote better hygiene practices to mitigate the spread of scabies.

## Supporting information

S1 Fig Schematic diagram of the sampling technique used to determine the prevalence and associated risk factors in the Hawassa Zuria District, Sidama Region, southern Ethiopia, 2023. (TIF)

S1 Appendix Questionnaire. (DOCX)

S1 Table Bivariate logistic regression analysis of factors associated with scabies in Hawassa Zuria District, southern Ethiopia, 2023. (DOCX)

## References

[1] Center for Disease Control and Prevention. Global Health, Division of Parasitic Diseases and Malaria. 2023. https://www.cdc.gov/parasites/scabies/ Accessed 6 Jun 2023.

[2] World Health Organization. Neglected tropical diseases. Scabies. 2023. www. Scabies (who.int) Accessed 31 May 2023.

[3] Karimkhani C, Colombara DV, Drucker AM, Norton SA, Hay R, Engelman D, et al. The global burden of scabies: a cross-sectional analysis from the Global Burden of Disease Study 2015. Lancet Infect Dis. 17(12):1247–54. doi: 10.1016/S1473-3099(17)30483-8 28941561 PMC5700804

[4] Federal Ministry of Health. Intrem-guideline for multi-sectorial scabies outbreak emergency response. Addis Ababa, Ethiopia; 2015. www.bing.com/ethiopia_guideline_for_Scabies_outbreak. Accessed 23 Jun 2024.

[5] Engelman. D, Yoshizumi J, Hay RJ, Osti M, Micali G, Norton S, et al. The 2020 International Alliance for the Control of Scabies Consensus Criteria for the diagnosis of Scabies. Br J Dermatol. 2020;183(5):808–20. doi: 10.1111/bjd.18943 32034956 PMC7687112

[6] Chandler DJ, Fuller LC. A review of scabies: an infestation more than skin deep. Dermatol. 2019;235:79–90. doi: 10.1159/000495290 30544123

[7] Galván-Casas C, Mitjà O, Esteban S, Kafulafula J, Phiri T, Navarro-Fernández Í, et al. A facility and community-based assessment of scabies in rural Malawi. PLoS Negl Trop Dis. 2021;15(6). doi: 10.1371/journal.pntd.0009386 34061851 PMC8195395

[8] Hay RJ, Steer AC, Engelman D, Walton S (2012) Scabies in the developing world-its prevalence, complications, and management. Eur Soc Clin Infect Dis 18(4): 313–323. doi: 10.1111/j.1469-0691.2012.03798.x 22429456

[9] Mohammed KA, Deb RM, Stanton MC, Molyneux DH. Soil-transmitted helminths and scabies in Zanzibar, Tanzania following mass drug administration for lymphatic filariasis—a rapid assessment methodology to assess impact. Parasit Vectors. 2012;5:299. doi: 10.1186/1756-3305-5-299 23259465 PMC3543323

[10] Ejigu K, Haji Y, Toma A, Tadesse BT. Factors associated with scabies outbreaks in primary schools in Ethiopia: a case-control study. Res Rep Trop Med. 2019;10:119–27. doi: 10.2147/RRTM.S214724 31695552 PMC6717729

[11] Henok D, Awrajaw D, Bikes D, Worku YW, Zemichael G. Prevalence and associated factors of scabies among schoolchildren in Dabat district, northwest Ethiopia, 2018. Environ Health Prev Med. 2019; doi: 10.1186/s12199-019-0824-6 31785612 PMC6885305

[12] Ugbomoiko US, Oyedeji SA, Babamale OA, Heukelbach J. Scabies in resource-poor communities in Nasarawa state, Nigeria: epidemiology, clinical features and factors associated with infestation. Trop Med Infect Dis. 3(2):13–5. doi: 10.3390/tropicalmed3020059 30274455 PMC6073861

[13] Currier RW, Walton SF, Currie BJ, Scabies in animals and humans: history, evolutionary perspectives, and modern clinical management. Ann N Y Acad Sci. 2012;1230(1):E50–60. 10.1111/j.1749-6632.2011.06364.x 22417107

[14] Ampem AY, Phillips RO, Arthur J, Abugri MA, Akowuah E, Amoako KO, et al. A scabies outbreak in the northeast region of Ghana: The necessity for prompt intervention. PLoS Negl Trop Dis. 2020; doi: 10.1371/journal.pntd.0008902 33351803 PMC7787682

[15] Kouotou EA, Nansseu JRN, Kouawa MK, Bissek AZ. Prevalence and drivers of human scabies among children and adolescents living and studying in Cameroonian boarding schools. Parasite Vectors. 2016; doi: 10.1186/s13071-016-1690-3 27430556 PMC4950090

[16] Hegab DS, Kato AM, Kabbash IA, Dabish GM. Scabies among primary schoolchildren in Egypt: sociomedical and environmental study in Kafr El-Sheikh administrative area. Clin Cosmet Investig Dermatol. 2015;8:105–11. doi: 10.2147/CCID.S78287 25759594 PMC4345923

[17] Engelman D, Cantey PT, Marks M, Solomon AW, Chang AY, Chosidow O, et al. The public health control of scabies: priorities for research and action. The Lancet. 2019;394: 81–92. doi: 10.1016/S0140-6736(19)31136-5 31178154 PMC11257500

[18] Azene AG, Aragaw AM, Wassie GT. Prevalence and associated factors of Scabies in Ethiopia: a systematic review and meta-analysis. BMC Infect Dis. 2020; doi: 10.1186/s12879-020-05106-3 32460770 PMC7254678

[19] Osti MH, Sokana O, Phelan S, Marks M, Whitfeld MJ, Gorae C, et al. Prevalence of scabies and impetigo in the Solomon Islands: a school survey. BMC Infect Dis. 2019; doi: 10.1186/s12879-019-4382-8 31519153 PMC6743115

[20] Abdel-Latif AA, Elshahed AR, Salama OA, Elsaie ML. Comparing the diagnostic properties of skin scraping, adhesive tape, and dermoscopy in diagnosing scabies. Acta Dermatovenerol Alp Pannonica Adriat. 2018;27(2):75–8. 29945263

[21] Walker SL, Lebas E, De Sario V, Deyasso Z, Doni N, Marks M, et al. The prevalence and association with health-related quality of life of tungiasis and scabies in schoolchildren in southern Ethiopia. PLoS Neglected Tropical Diseases. 2017;11: 1–11. doi: 10.1371/journal.pntd.0005808 28771469 PMC5557602

[22] Enbiale W, Ayalew A. Investigation of a Scabies outbreak in drought-affected areas in Ethiopia. Trop Med Infect Dis. 2018;3(4):114. doi: 10.3390/tropicalmed3040114 30380650 PMC6306922

[23] Girma E, Churko C, Alagaw A, Haftu D, Tunje A. Prevalence of Scabies and its associated factors among school-age children in Arba Minch Zuria District, southern Ethiopia. Preprint. 2020;.

[24] Sara J, Haji Y, Gebretsadik A. Scabies outbreak investigation and risk factors in east Badewacho District, southern Ethiopia: unmatched case-control study. Dermatol Res Pract. 2018; doi: 10.1155/2018/7276938 30046302 PMC6038489

[25] Ararsa G, Merdassa E, Shibiru T, Etafa W. Prevalence of scabies and associated factors among children aged 5–14 years in Meta Robi district, Ethiopia. PLoS One. 2023; doi: 10.1371/journal.pone.0277912 36595503 PMC9810185

[26] Worku ED, Asemahagn MA, Endalifer ML. Determinants of scabies outbreak in Takusa District of Amhara Region, northwest Ethiopia. J Public Health Africa. 2020;11(1325):122–6. 10.4081/jphia.2020.1325 PMC7893313 33623653

[27] Haile T, Sisay T, Jemere T. Scabies and its associated factors among under 15 years children in Wadila District, northern Ethiopia,2019. Pan-African Med J. 2020; doi: 10.11604/pamj.2020.37.224.25753 33520063 PMC7821794

[28] Wochebo W, Haji Y, Asnake S. Scabies outbreak investigation and risk factors in Kechabira District, southern Ethiopia: unmatched case-control study. BMC Res Notes. 2019; doi: 10.1186/s13104-019-4317-x 31142358 PMC6542071

[29] Tefera BN, Kim Y. Ethnobotanical study of medicinal plants in the Hawassa Zuria District, Sidama zone, Southern Ethiopia. J Ethnobiol Ethnomed. 2019;15(25):1–21. doi: 10.1186/s13002-019-0302-7 31126296 PMC6534827

[30] Likert R. “A technique for the measurement of attitudes,” Arch Psychol. 1932;22:5–55.

[31] Mm Birjandi, Oroei M Emadi Sn, Aa Peyvandi, Ak Anang. Scabies Among High School Students in Accra, Ghana: Risk Factors and Health Literacy. Iran Red Crescent Med J. 2019; 21(8): doi: 10.5812/Ircmj.92510

[32] Walter B, Heukelbach J, Fengler G, Worth C, Hengge U, Feldmeier H. Comparison of Dermoscopy, Skin scraping, and the Adhesive Tape test for the diagnosis of Scabies in a resource-poor setting. Arch Dermatol. 2011;147(4):468–73. doi: 10.1001/archdermatol.2011.51 21482897

[33] EDHS. Central Statistical Agency (CSA) [Ethiopia] and ICF. Ethiopia Demographic and Health Survey 2016. Addis Ababa, Ethiopia, and Rockville, Maryland, USA: CSA and ICF. 2017.

[34] Croft, Trevor N., Aileen M. J. Marshall, Courtney K. Allen, et al. Guide to DHS Statistics. Rockville, Maryland, USA: ICF. 2018.

[35] WHO. Integrating water quality testing into household survey: thematic report on drinking water. New York: United Nations Children’s Fund and World Health Organization; 2020.

[36] Hosmer DW Jr, Lemeshow S, Sturdivant RX. Applied logistic regression. 3rd ed. Hoboken: John Wiley & Sons; 2013.

[37] Amare HH, Bernt L. Risk factors for scabies, tungiasis, and tinea infections among schoolchildren in southern Ethiopia: a cross-sectional Bayesian multilevel model. PLoS Negl Trop Dis. 2021; doi: 10.1371/journal.pntd.0009816 34613968 PMC8494366

[38] Fischer K, Holt D, Currie B, Kemp D. Scabies: important clinical consequences explained by new molecular studies. Adv Parasitol. 2012;79:339–73. doi: 10.1016/B978-0-12-398457-9.00005-6 22726646

[39] Liu J, Wang H, Chang F, Liu Y, Chiu F. The effects of climate factors on scabies. A 14-year population-based study in Taiwan. parasite. 2016; doi: 10.1051/parasite/2016065 27905271 PMC5134670

[40] Shelui C, Timothy J, Zayzay SK, Kollie KK, Lebas E, Candy N, et al. The prevalence of scabies in Monrovia, Liberia: A population-based survey. PLoS Negl Trop Dis. 2020; doi: 10.1371/journal.pntd.0008943 33284821 PMC7746289

[41] Walton SF, Currie BJ. Problems in Diagnosing Scabies, a global disease in human and animal populations. Clin Microbiol Rev. 2007;20(2):268–79. doi: 10.1128/CMR.00042-06 17428886 PMC1865595

[42] Sanei-dehkordi A, Soleimani-Ahmadi M, Zare M, Jaberhashemi SA. Risk factors associated with scabies infestation among primary schoolchildren in a low socioeconomic area in southeast Iran. BMC Pediatr. 2021; doi: 10.1186/s12887-021-02721-0 34034686 PMC8145826

[43] Arlian LG, Morgan MS. A review of Sarcoptes scabiei: past, present and future. Parasite Vectors. 2017; doi: 10.1186/s13071-017-2234-1 28633664 PMC5477759

[44] Romani L, Koroivueta J, Steer AC, Kama M, Kaldor JM, Wand H, et al. Scabies and impetigo prevalence and risk factors in Fiji: A national survey. PLoS Negl Trop Dis. 2015; doi: 10.1371/journal.pntd.0003452 25738499 PMC4349858

[45] Gowtham V, Kalyani P, Felix AJW, Felix AJW. Prevalence of scabies among school children living in urban Chidambaram and its associated risk factors: a cross-sectional study. Int J Community Med Public Health. 2020;7(12):4879–84.

[46] Feldmeier H, Jackson A, Ariza L, Lins Calheiros CM, De Lima Soares V, Oliveira FA, et al. The epidemiology of scabies in an impoverished community in rural Brazil: Presence and severity of disease are associated with poor living conditions and illiteracy. J Am Acad Dermatol. 2009;60(3):436–43. doi: 10.1016/j.jaad.2008.11.005 19064303

[47] Misganaw B, Nigatu SG, Gebrie GN, Anteneh AK. Prevalence and determinants of scabies among school-age children in Central Armachiho District. northwest Ethiopia. PLoS One. 2022; doi: 10.1371/journal.pone.0269918 35700176 PMC9197047

[48] Cinotti E, Perrot JL, Labeille B, Maguet H, Couzan C, Flori P, et al. Inefficacy of alcohol-based hand rub on mites in a patient with hyperkeratotic scabies. Clin Exp Dermatol. 2015;40(2):177–81. doi: 10.1111/ced.12467 25251891

[49] Walton SF, Pizzutto S, Slender A, Viberg L, Holt D, Hales BJ, et al., Increased allergic immune response to Sarcoptes scabiei antigens in crusted versus ordinary scabies. Clin Vaccine Immunol. 17(9):1428–38. doi: 10.1128/CVI.00195-10 20631334 PMC2944463

