# Supplementary figures and images for: Assessment of scabies and its associated factors in Hawassa Zuria District, Southern Ethiopia: A cross-sectional study
Source: PLoS One. 2024 Nov 21;19(11):e0314140. doi: 10.1371/journal.pone.0314140 (PMC11581355; doi:10.1371/journal.pone.0314140)

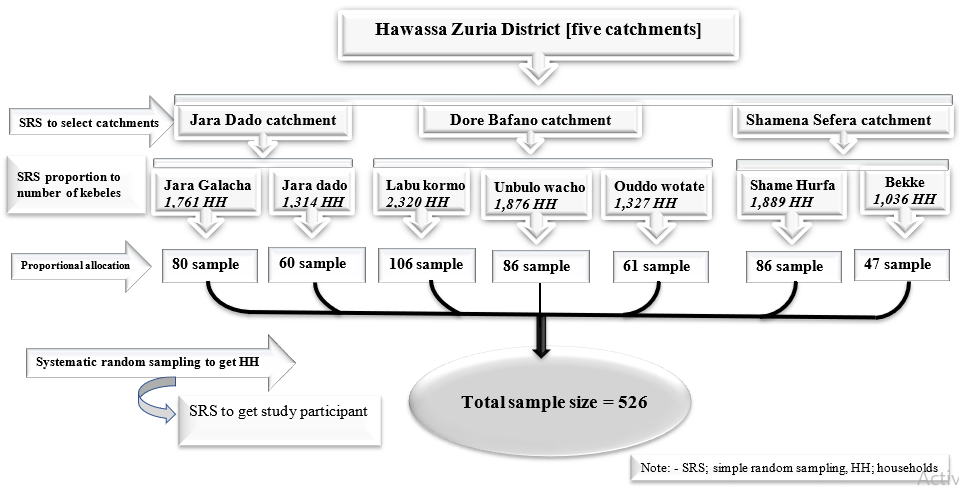

Supplement: S1 Fig — (TIF) [file pone.0314140.s001.tif]
