# Supplementary material for: Assessment of scabies and its associated factors in Hawassa Zuria District, Southern Ethiopia: A cross-sectional study
Source: PLoS One. 2024 Nov 21;19(11):e0314140. doi: 10.1371/journal.pone.0314140 (PMC11581355; doi:10.1371/journal.pone.0314140)
Supplement: S1 Appendix — (DOCX) [file pone.0314140.s002.docx]

**S1 Appendix: QUESTIONNAIRE (ENGLISH VERSION)**

**General Instructions**: - Please ask the responder each question clearly and carefully, then encircle the number (coding category), if the participant mentions single or multiple responses among the given options in front of each question. For each open-ended question, fill in the blank spaces on the right side of each question with the best description provided by the interviewee. Any participant’s response not in the options list would be captured under the option “If others (specify)”. Please do not read the options for participants unless specified. **(**All the questionnaires are to be completed by health officers)

| Part one: Questionnaires regarding participant’s socio-demographic and wealth index characteristics | | | |
| --- | --- | --- | --- |
| Participant identification number (code) ______________Date______/_______/_______*Kebele* _______ | | | |
| No | Questions | Coding category and possible responses /answers | |
|  | How old are you? | \|  \| \| --- \|   (Age in years) | |
|  | What is your Gender (sex)? | 1. Male | 1. Female |
|  | Where do you live (address)? | 1. Urban | 1. Rural |
|  | Do you attain formal education?  What is the highest educational level you attended? | 1. No formal education and unable to Read/write 2. No formal schooling but can Read/write 3. Primary education (Grades 1-4) 4. Primary education (Grades 5-8) 5. Secondary education (Grades 9-10) 6. Preparatory education (Grade 11-12) 7. Collage and above | |
|  | What is your occupation? | 1. Farmer 2. Student 3. Housewife 4. Merchant 5. Daily Laborer | 1. Civil servant 2. Private employee 3. Jobless 4. If others (specify) ____? |

*************************************************************************************

|  | **General information about study participants' household characteristics**  To fill the next part of the questionnaire I need to observe what materials you have used to build the floor, wall, and roof of your house. Is it ok if I take a walk with you in the compound and do the observations? | | | | | | | |  |
| --- | --- | --- | --- | --- | --- | --- | --- | --- | --- |
|  | Are you the Owner of the house? | 1. Yes (Private) 2. No (Rented) | | | 1. If others (specify) ___? | | | |  |
|  | What are the main materials of the Roof of the dwelling (Note: Observe and record one answer, if more than one tick the one covers a large space) | 1. Tiles 2. Corrugated Iron 3. Cement 4. Reed/Bamboo | | | 1. Mud 2. Thatch 3. Plastic 4. If others (specify) ___? | | | |  |
|  | What are the main materials of the wall of the dwelling (Note: Observe and record one answer, if more than one tick the one covers a large space) | 1. Cement 2. Wood Planks/Shingles 3. Bricks | | | 1. Mud 2. Palm/Trunks/Bamboo 3. If others (specify)___? | | | |  |
|  | What are the main materials of the floor of the dwelling? (Note: Ask permission to observe the facility and record one answer, if more than one tick the one covers a large space) | 1. Cement 2. Ceramic 3. Bricks 4. Carpet | | | 1. Earth/Sand 2. Dung 3. Wood plank 4. If Others (Specify) __? | | | |  |
|  | What type of fuel does your household mainly use for cooking? | 1. Electricity 2. Gas 3. Charcoal | | | 1. Animal dung 2. Wood 3. If Others (Specify) __? | | | |  |
|  | What kind of toilet facility do members of your household usually use?  (Ask permission to observe the facility) | 1. Flush/pour flush toilet 2. Ventilated Improved pit Latrine | | | 1. Pit latrine with slab 2. Pit latrine without slab (Open Pit) 3. None (Bush Field) 4. If Others (Specify) __? | | | |  |
|  | Do you share this toilet facility with other households? | | 1. Yes | | | | 1. No | |  |
|  | Do you have a separate room which is used as a kitchen? | | 1. Yes | | | | 1. No | |  |
|  | **II. Household asset:** Now I would like to ask you a few questions about the assets your household owns, this information helps us to assess the contribution of household wealth to the health status of members of the household | | | | | | | |  |
|  | Does your household have: | | | | | | | |  |
|  | 1. A radio? | | 1. Yes | | | 1. No | |  |  |
|  | 1. A refrigerator? | | 1. Yes | | | 1. No | |  |  |
|  | 1. A computer? | | 1. Yes | | | 1. No | |  |  |
|  | 1. A non-mobile telephone? | | 1. Yes | | | 1. No | |  |  |
|  | 1. A television? | | 1. Yes | | | 1. No | |  |  |
|  | 1. A chair? | | 1. Yes | | | 1. No | |  |  |
|  | 1. A table? | | 1. Yes | | | 1. No | |  |  |
|  | 1. A bed with cotton/ sponge/spring matter? | | 1. Yes | | | 1. No | |  |  |
|  | 1. An electric Mitad? | | 1. Yes | | | 1. No | |  |  |
|  | 1. A kerosene lamp/pressure lamp | | 1. Yes | | | 1. No | |  |  |
|  | Does any member of this household own? | | | | | | |  |  |
|  | 1. A watch/clock? | | 1. Yes | | | 1. No | |  |  |
|  | 1. A Bagag | | 1. Yes | | | 1. No | |  |  |
|  | 1. A mobile phone? | | 1. Yes | | | 1. No | |  |  |
|  | 1. A bicycle? | | 1. Yes | | | 1. No | |  |  |
|  | 1. A motorcycle or motor scooter? | | 1. Yes | | | 1. No | |  |  |
|  | 1. An animal-drawn cart? | | 1. Yes | | | 1. No | |  |  |
|  | 1. A car or truck? | | 1. Yes | | | 1. No | |  |  |
|  | 1. A boat with a motor? | | 1. Yes | | | 1. No | |  |  |
|  | Does any member of this household have a bank account or microfinance savings account? | | 1. Yes | | | 1. No | |  |  |
|  | Does the household own agricultural land? | | 1. Yes | | | 1. No | |  |  |
|  | If Q 12 is answered yes, how many hectares of agricultural land do members of this household own? | | -----------------(hectares) | | | | |  |  |
|  | Does this household own any livestock, herds, other farm animals, or poultry? 1. Yes 2. No | | | | | | | | |
|  | How many of the following animals does this Household own | | | | | | | | |
|  | 1. Milk cows, oxen, or bulls? | | | ------------- in Number | | | |  |  |
|  | 1. Horses, donkeys, or mules? | | | ------------- in Number | | | |  |  |
|  | 1. Goats? | | | ------------- in Number | | | |  |  |
|  | 1. Sheep? | | | ------------- in Number | | | |  |  |
|  | 1. Chickens or other poultry? | | | ------------- in Number | | | |  |  |
|  | 1. Bee hives? | | | ------------- in Number | | | |  |  |
|  | 1. Other cattle? | | | ------------- in Number | | | |  |  |

*************************************************************************************

| Part two: Questionnaires regarding participant’s Behavioral and Environmental-related factors | | | | | |
| --- | --- | --- | --- | --- | --- |
| No | Questions | | Coding and possible responses | | |
|  | How many Persons live together in the house? | \|  \| \| --- \|   Individuals | | | |
|  | How many sleeping rooms (beds) do the family members have? | \|  \| \| --- \|   Rooms | | | |
|  | Where do you Sleep? | 1. On bed | | | 1. On the floor |
|  | Do you Sleep with family members (bed sharing)? | 1. Yes | | | 1. No |
|  | Do you share a blanket during bedtime? | 1. Yes | | | 1. No |
|  | Have you shared your clothes with a person with scabies in the last 2 months? | 1. Yes | | | 1. No |
|  | Have you had contact with a scabies-infested person (skin itching case) in the last 2 months? | 1. Yes | | | 2. No🡪 Go to Q 209 |
|  | If Q 301 is yes, his/ her relationship with you? | 1. Family member. 2. Neighbors 3. Class-mate | | | 1. Work-mate 2. Other (specify)----------- |
|  | Is there a family member's history of scabies (itching) in the last two months? | 1. Yes | | | 2. No |
|  | What kind of climatic zone is a respondent living in? | 1. Lowland 2. Midland | | | 1. High land |
|  | Do pet animals live inside the house with people? | 1. Yes | | | 2. No 🡪 Go to Q 212 |
|  | If Q 208 is yes, specify the name of the animal. | \|  \| \| --- \| | | | |
|  | Is there any flooding in your area? | 1. Yes | | 2. No 🡪 Go to Q 213 | |
|  | If Q 211 is answered yes, is your home affected by flooding this year? | 1. Yes | | 2. No | |
|  | How much time it takes to go to the nearest health facility? | \|  \| \| --- \|   minutes | | | |

*************************************************************************************

| Part three: Questionnaires regarding participant’s personal hygiene and sanitation practice | | | |
| --- | --- | --- | --- |
| No | Questions | Coding category and possible responses /answers | |
| 1. 3 | Do you have access to health education on personal hygiene? | 1. Yes | 1. No |
|  | When do you clean your house? | 1. Every day 2. Every other day 3. Once per week | 1. Once per 2 weeks 2. Once per Month 3. Not at all 4. If others (specify) ___? |
|  | What do you mainly (regularly) use during your hand wash? | 1. Water only 2. Soap and water | 1. Water and ash 2. If others (specify) ___? |
|  | How frequently do you wash your body? | 1. Every day 2. Every other day 3. Once per week | 1. Once per 2 weeks 2. Once per Month 3. Not at all 4. If others (specify) ___? |
|  | How frequently do you wash your clothes? | 1. Every day 2. Every other day 3. Once per week | 1. Once per 2 weeks 2. Once per Month 3. Not at all 4. If others (specify) ___? |
|  | How frequently do you change your clean clothes? | 1. Every day 2. Every other day 3. Once per week | 1. Once per 2 Weeks 2. Once per Month 3. Not at all 4. If others (specify) ___? |
|  | How frequently do you wash your hair? | 1. Every day 2. Every other day 3. Once per week | 1. Once per 2 weeks 2. Once per month 3. Not at all 4. If others (specify) ___? |
|  | Does the participant cut (trim) his/her fingernails? (Observe and record) | 1. Yes | 1. No |
|  | What is the main source of water for personal hygiene for the members of your HH? | 1. Pipe Water 2. Protected spring 3. Protected well 4. Rainwater 5. Unprotected well 6. Unprotected spring | 1. Tanker truck-provided water 2. Surface water (river/dam/lake/pond/stream/canal 3. If others (specify) ___? |
|  | Do you have the habit of Ironing/drying clothes and bedding in the sun | 1. Yes 2. No | |
|  | How much time does it take to fetch water? (Time to go there, get water, and come back?) | \|  \| \| --- \|   Minute | |
|  | How many liters of water does the HH consume per day for domestic purposes (estimated)? | \|  \| \| --- \|   Liters | |

*************************************************************************************

| Part four: Questionnaires regarding the participant’s, or the participant's parents/guardian's General Knowledge of scabies  I will ask you a few questions about your knowledge of scabies. You will just tell me what you know about the disease, it doesn’t need to be the right answer. | | | | |
| --- | --- | --- | --- | --- |
| No | Questions | Coding category and possible responses /answers | | |
|  | Have you ever heard about scabies disease before? | 1. Yes | 1. No 🡪 Go to Q 403 | |
|  | If yes to Q 401 What is your source of information? | 1. Radio 2. Magazine 3. Television 4. Health workers | 1. School 2. Religious leaders 3. Social media 4. If others (specify) ____? | |
|  | What is the Etiology agent of scabies? | 1. Parasite 2. witchcraft 3. God’s anger 4. Curse | 1. The effect of scratching 2. Do not know 3. If others (specify) ____? | |
|  | How scabies is transmitted from person to person? (Note: Multiple responses are possible) | 1. Via skin-to-skin contact with infected person 2. Through clothes and bed-sharing 3. Through Mosquito bite 4. Via blood contact 5. By Contact with pet animal 6. Through drinking contaminated water 7. I do not know the mode of transmission 8. If others (specify) ____? | | |
|  | How can we prevent (break) scabies transmission? (Note: Multiple responses are possible) | 1. Giving Treatment to infected persons 2. Avoiding physical contact with scabies patient 3. By avoiding sharing of clothes and bedding 4. Visiting a health facility when feeling sick 5. Through improving hygiene and sanitation 6. Do not know 7. If others (specify) ____? | | |
|  | What is/are the symptoms of scabies? (Note: Multiple responses are possible) | 1. Itching 2. Skin Rash | | 1. Do not know 2. If others (specify) ___? |
|  | Does Scabies be harmful to the health of the skin? | 1. Yes | | 1. No |
|  | Does ironing or drying of bedding (mattresses, blankets, and pillows) in the sun reduce scabies transmission? | 1. Yes | | 1. No |
|  | Which age groups are more affected by scabies? (Note: Multiple responses are possible) | 1. Children 2. Adults | | 1. All age groups 2. Elders |
|  | Is scabies a curable (treatable) disease? | 1. Yes | | 2. No |

*************************************************************************************

| **Part five: Questionnaires regarding participant’s attitudes about scabies**  **Instruction**: Please ask each question and tick (“✓”) the degree of agreement in the provided box in front of each question with respect to each possible response in the Likert scale form instructed under the coding category. | | | | | | |
| --- | --- | --- | --- | --- | --- | --- |
| No | Questions | Coding category and possible responses /answers | | | | |
|  |  | Strongly Disagree (1) | Disagree (2) | Neutral (3) | Agree (4) | Strongly Agree (5) |
|  | I believe that it is important to confirm a scabies diagnosis at a health facility before treatment. |  |  |  |  |  |
|  | I feel compassion and wish to help scabies-infested individuals without exposing myself. |  |  |  |  |  |
|  | I’m sure that self-treatment may endanger my health |  |  |  |  |  |
|  | I’m not interested in sharing my clothes with scabies-infested individuals. |  |  |  |  |  |
|  | I believe that the most effective approach to keeping me from acquiring scabies is to avoid skin-to-skin contact. |  |  |  |  |  |
|  | I believe that maintaining proper personal hygiene is one way to protect myself from Scabies. |  |  |  |  |  |
|  | I’m sure that everyone can be infested with scabies. |  |  |  |  |  |
|  | In my opinion, individuals in the marginal age are more vulnerable to scabies. |  |  |  |  |  |
|  | I will seek medical advice if I become infested with scabies. |  |  |  |  |  |
|  | I feel shame/fear if I become infested with scabies. |  |  |  |  |  |
|  | I think that infestation with scabies may facilitate secondary bacterial infection. |  |  |  |  |  |

************************* ************************************************************

| Part six: Questionnaires regarding participant’s clinical examination and laboratory diagnosis  **Instructions**: Data collectors should examine the entire body of the participant and record what he/she observes. Any participant’s response not in the options list would be captured under the option “If others (specify)”. | | | | | | | | | | |
| --- | --- | --- | --- | --- | --- | --- | --- | --- | --- | --- |
| No | | | Questions | | | Coding category and possible responses /answers | | | | |
|  | | Do the participants have a visible lesion? | | | | 1. Yes | | | 2. No | |
|  | | If Q 601 answer is Yes. How many lesions are seen in the infested body part? | | | | \|  \| \| --- \| | | | | |
|  | | What type of lesion is seen in the body of an infested individual? | | | | 1. Papules 2. Nodular lesion 3. pustules | | | 1. vesicles 2. crusts on the skin 3. If others (specify) ____? | |
|  | | If Q 601 answer is Yes. Which body part is affected?  (Note: multiple responses possible) | | | | 1. Flexor wrist surface 2. Abdomen 3. Inter-digital Spaces 4. Buttocks 5. Lower leg | | | 1. Feet 2. Elbow 3. Armpit 4. Shoulder blades 5. If others (specify) ____? | |
|  | | What are the Clinical features (signs) of scabies?  (Note: multiple responses possible) | | | | 1. Red bumps and blisters 2. Tiny red burrows 3. Crusts on the skin | | | 1. Skin rash 2. Itching 3. If others (specify) ____? | |
|  | | If the Q 604 answer is Itching, at what time is the itching intense? | | | | 1. Day Time 2. Night Time | | | 1. Similar in both day and night | |
|  | | Result of physical examination based on IACS criteria | | | | ___________________________________ | | | | |
|  | | Result of the Microscopic examination | | | | ___________________________________ | | | | |
|  | | |  |  | |  |  | |  |  |
| Name Of Interviewer | | |  | Signature | |  | Date | |  |  |

**Thank you very much!**
