# Supplementary material for: Assessment of scabies and its associated factors in Hawassa Zuria District, Southern Ethiopia: A cross-sectional study
Source: PLoS One. 2024 Nov 21;19(11):e0314140. doi: 10.1371/journal.pone.0314140 (PMC11581355; doi:10.1371/journal.pone.0314140)
Supplement: S1 Table — (DOCX) [file pone.0314140.s003.docx]

| **Variables** | **Category** | **Scabies diagnosis** | | **COR (95% CI)** | **P - value** |
| --- | --- | --- | --- | --- | --- |
|  |  | **Yes (%)** | **No (%)** |  |  |
| Gender | Female | 11 (4.0) | 259 (96.0) | 1 |  |
|  | Male | 21 (8.7) | 220 (91.3) | 2.59 (1.20-5.58) | **0.015*** |
| Age | <5 | 3 (6.5) | 43 (93.5) | 1 |  |
|  | 5-9 | 5 (8.6) | 53 (91.4) | 1.35 (0.30-5.98) | 0.691 |
|  | 10-14 | 8 (8.3) | 88 (91.7) | 1.30 (0.32-5.15) | 0.706 |
|  | 15-24 | 9 (7.8) | 107 (92.2) | 1.20 (0.31-4.66) | 0.787 |
|  | 25-34 | 3 (4.0) | 73 (96.0) | 0.58 (0.11-3.04) | 0.528 |
|  | 35-44 | 1 (1.8) | 56 (98.2) | 0.25 (0.02-2.54) | **0.245*** |
|  | ≥45 | 3 (4.8) | 59 (95.2) | 0.72 (0.14-3.78) | 0.707 |
| Educational status | No formal education | 9 (6.1) | 139 (93.9) | 0.74 (0.25-2.18) | 0.59 |
|  | Primary school | 17 (5.9) | 271(94.1) | 0.72 (0.27-1.90) | 0.51 |
|  | Secondary & above | 6 (8) | 69 (92) | 1 |  |
| Wealth index | High | 3 (1.8) | 167 (98.2) | 1 |  |
|  | Middle | 17 (9.4) | 154 (90.6) | 5.78 (1.65-20.23) | **0.006*** |
|  | Low | 13 (7.6) | 158 (92.4) | 4.58 (1.28-16.38) | **0.019*** |
| Overcrowding index | $\leq$1.5 | 10 (3.3) | 297 (96.7) | 1 |  |
|  | >1.5 | 22 (10.8) | 188 (89.2) | 3.59 (1.66-7.75) | **0.001*** |
| The presence of flooding in your area | Yes | 12 (7.2) | 154 (92.8) | 1.27 (0.60-2.66) | 0.532 |
|  | No | 20 (5.8) | 325 (94.2) | 1 |  |
| Homes affected by Flooding | Yes | 1 (3.7) | 26 (96.3) | 0.45 (0.06-3.62) | 0.451 |
|  | No | 11 (7.9) | 128 (92.1) | 1 |  |
| Distance of health facility from the household | Near (≤1hours) | 24 (6.8) | 330 (93.2) | 1 |  |
|  | Far (>1 hours) | 8 (5.1) | 149 (94.9) | 0.74 (0.32-1.68) | 0.470 |
| The climatic condition of the participant’s residence site | Low land | 16 (4.6) | 330 (95.4) | 1 |  |
|  | Midland | 16 (9.7) | 149 (90.3) | 2.21 (1.07-4.54) | **0.030*** |
| Distance of water source from household | Basic service (≤30’) | 16 (6.0) | 250 (94.0) | 1 |  |
|  | Limited service (>30’) | 16 (6.5) | 229 (93.5) | 1.09 (0.53-2.23) | 0.810 |
| The presence of pet animals in the house | Yes | 11 (5.5) | 189 (94.5) | 0.80 (0.38-1.71) | 0.569 |
|  | No | 21 (6.8) | 290 (93.2) | 1 |  |
| Access to health education | Yes | 10 (4.8) | 218 (95.2) | 1 |  |
|  | No | 22 (7.8) | 261 (92.2) | 1.83 (0.85-3.96) | **0.121*** |
| Hand washing practice | Soap and water | 12 (4.4) | 286 (95.6) | 1 |  |
|  | With water only | 20 (9.4) | 193 (90.6) | 2.46 (1.18-5.16) | **0.016*** |
| Approximate amount of water accessed by households | Insufficient (≤30 liter) | 15 (7.2) | 192 (92.8) | 1.32 (0.64-2.70) | 0.450 |
|  | Sufficient (>30 liter) | 17 (5.6) | 287 (94.4) | 1 |  |
| Source of water for personal hygiene | Improved | 17 (5.1) | 318 (94.9) | 1 |  |
|  | Unimproved | 15 (10.1) | 161 (89.9) | 1.74 (0.84-3.57) | **0.130*** |
| Bathing frequency | Frequent | 16 (5.1) | 298 (94.9) | 1 |  |
|  | Infrequent | 16 (8.5) | 181 (91.5) | 1.64 (0.80-3.37) | **0.173*** |
| Clothes washing frequency | Frequent | 13 (5.2) | 238 (94.8) | 1 |  |
|  | Infrequent | 19 (7.3) | 241 (92.7) | 1.44 (0.70-2.99) | 0.323 |
| Frequent changing of clothes | Yes | 17 (6.3) | 254 (93.7) | 1 |  |
|  | No | 15 (6.2) | 225 (93.8) | 0.99 (0.49-2.04) | 0.991 |
| Hair washing frequency | Frequent | 20 (6.8) | 272 (93.2) | 1 |  |
|  | Infrequent | 12 (5.5) | 207 (94.5) | 0.79 (0.38-1.65) | 0.528 |
| Fingernail trimming | Yes | 22 (6.8) | 301 (93.2) | 1 |  |
|  | No | 10 (5.3) | 178 (94.7) | 0.77 (0.36-1.66) | 0.503 |
| Personal hygiene practice | Good | 15 (6.7) | 210 (93.3) | 1 |  |
|  | Poor | 17 (5.9) | 269 (94.1) | 0.88 (0.43-1.81) | 0.738 |
| Sleeping place | On the bed | 12 (3.8) | 307 (96.2) | 1 |  |
|  | On the floor | 20 (10.4) | 172 (89.6) | 2.97 (1.41-6.23) | **0.004*** |
| Bed sharing | No | 9 (4.9) | 176 (95.1) | 1 |  |
|  | Yes | 23 (7.1) | 303 (92.9) | 1.48 (0.67-3.28) | 0.329 |
| Cloth sharing with scabies cases | No | 29 (5.9) | 467 (94.1) | 1 |  |
|  | Yes | 3 (20.0) | 12 (80.0) | 4.02 (1.07-15.06) | **0.039*** |
| Contact with scabies case | No | 27 (5.7) | 451 (94.3) | 1 |  |
|  | Yes | 5 (15.2) | 28 (84.8) | 2.98 (1.06- 8.33) | **0.037*** |
| A habit of drying clothing/bedding in the sun | Yes | 21 (6.2) | 320 (93.8) | 1 |  |
|  | No | 11 (6.5) | 159 (93.5) | 1.05 (0.50-2.24 | 0.891 |
| Family member with scabies | No | 28 (5.8) | 458 (94.2) | 1 |  |
|  | Yes | 4 (16.0) | 21 (84.0) | 3.11 (1.00-9.69) | **0.050*** |
| Mean knowledge score | Good knowledge | 19 (5.8) | 307 (94.2) | 1 |  |
|  | Poor knowledge | 13 (8.0) | 149 (92.0) | 1.41 (0.68-2.93) | 0.358 |
| Overall attitude score | Negative attitude | 12 (8.2) | 134 (91.8) | 1.44 (0.68-3.03) | 0.335 |
|  | Positive attitude | 20 (5.8) | 322 (94.2) | 1 |  |

Note: - * statistically significant at p-value < 0.25, COR Crude Odds Ratio, CI Confidence interval
